# A Model of Stress-Damage-Permeability Relationship of Weakly Cemented Rocks under Triaxial Compressive Conditions

**DOI:** 10.3390/ma16010210

**Published:** 2022-12-26

**Authors:** Shizhong Zhang, Gangwei Fan, Dongsheng Zhang, Wenping Li, Tao Luo, Shuaishuai Liang, Zhanglei Fan

**Affiliations:** 1 School of Mines, China University of Mining & Technology, Xuzhou 221116, China; 2 School of Resources and Earth Science, China University of Mining & Technology, Xuzhou 221116, China; 3 School of Mines, Saint Petersburg Mining University, 199106 Saint Petersburg, Russia

**Keywords:** weakly cemented rocks, triaxial compression, permeability, matrix fracturing, stress–damage–permeability

## Abstract

To unravel the permeability variation mechanism of weakly cemented rocks (WCR), the paper conducted triaxial permeability tests on weakly cemented sandstones (WCS) collected from the Jurassic formation in northwest China. The paper identified the correlation of WCS permeability versus porosity, cementation structure, and mineral composition, further developing a model to characterize the WCS stress–damage–permeability relationship. The research indicated that the WCS permeability was initially high due to the naturally high porosity, large pore diameter, and loose particle cementation, thus favoring a significant decline as pore convergence in the compaction stage. In the residual stage, kaolinite and montmorillonite minerals disintegrated into water and narrowed fractures, causing a slight permeability increase from the initial to the maximum and residual stages. The WCS matrix fracturing was phenomenologically accompanied by clay mineral disintegration. By assuming that the matrix can be compressed, jointed, and fractured, the paper defined a damage variable *D* and accordingly developed a stress–damage–permeability relationship model that incorporated matrix compression, jointing, and fracturing. The model can describe the WCS permeability regime regarding the high initial permeability and slight difference of the maximum and residual permeabilities versus the initial.

## 1. Introduction

Weakly cemented rocks (WCR) widely exist in nature and contribute to the impact of geotechnical projects on the localized water system and ecological environment. Understanding WCR permeability responses to different stress conditions helps provide guidance for operating hydropower, underground tunneling, and mine projects [1,2,3,4]. In northwest China, the rock units in Jurassic and Cretaceous formations are often weakly cemented, generally featuring young sedimentation, low strength, and loose cementation. Such properties cause weakly cemented rocks to be sensitive to engineering perturbations [5,6]. Moreover, the arid and semi-arid climate, water resource scarcity, and eco-environmental fragility in northwest China further aggravate the impact of WCR permeability increase on groundwater depressurization and thus environmental deterioration [7,8]. Therefore, understanding the WCR permeability variation mechanism is of great significance for both geotechnical practice and eco-environmental protection. 

The current progress of weakly cemented rocks is mainly in rock properties and permeability [9,10,11]. In rock mechanics, Sharma and Wang et al. conducted laboratorial tests and quantified the correlation of bulk density and P-wave velocity versus axial compressive strength and elastic modulus of weakly cemented mudstone (WCM) and sandstone (WCS) [12,13]. Konstantinou et al. analyzed the relationship of WCS tensile strength with fracture patterns, surface features, and cementing degree [14]. Li and Zhao et al. studied the stress–strain, acoustic emission, and energy evolution characteristics of weakly cemented rocks under Brazilian test conditions, by which their relationship was discussed [15,16]. Liu et al. conducted triaxial compression tests and analyzed the impact of water content and confining pressure on fracture pattern and stress–strain curves of WCM and WCS samples [17]. Tommasi and Ye et al. proposed that WCR mechanical behavior is correlated to physical and structural properties, further analyzing the impact of pore abundance, mineral composition, and matrix-pore continuity on rock strength [18,19]. Regarding WCR hydraulics, Nguyen and Ruistuen et al. analyzed the impact of the stress path on WCS permeability and identified that the permeability had a significant decline when the shear strength was exceeded, which was attributed to particle friction and pore tortuosity increase [20,21]. You et al. and Liu et al. analyzed the impact of temperature, confining pressure, and water content on WCR permeability and developed a fractional quaternion model to describe WCR creep behavior in three continuous stages [22,23]. Liu et al. analyzed WCS permeability variation with porosity and mercury injection rate, by which the pore–throat ratio was considered a major indicator of permeability mutation [24]. Lyu et al. conducted laboratorial tests and analyzed WCS permeability features with varying particle diameters, revealing that the permeability coefficient was positively correlated to particle diameter in the same confining pressure and hydraulic pressure condition, between which the pore water pressure had greater impacts [25].

Previous research has investigated WCR strength properties and tried qualitative analysis on the permeability variation from physical perspectives, consolidating the basis for this paper. According to knowledge from geotechnical practices, the microstructural damage induced by stress environment change should be the critical contribution to WCR permeability variation. In this context, the paper collected WCR samples from the Jurassic formation in Ili mine fields, China and conducted complete stress–strain permeability tests using Rock Top multi-physics platform. The paper further studied the impact of porosity feature, cementation structure, and mineral composition on permeability variation. A WCR representative element volume (REV) model was constructed by considering physical mechanics and permeability property, by which, coupled with defining a damage variable *D*, a WCR stress–damage–permeability relationship model was developed. This research can provide theoretical reference for geotechnical operation and eco-environmental protection in a weakly cemented rock environment.

## 2. Materials

### 2.1. Sampling

**Figure 1:**
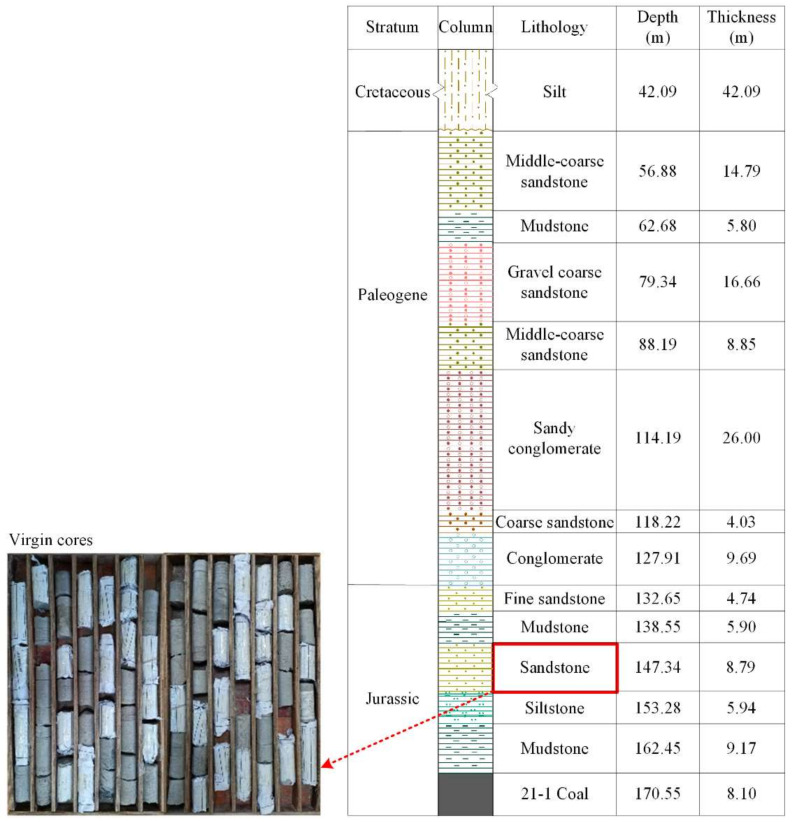
Borehole profile and WCS sampling position.

The research first collected WCS samples from the Middle Jurassic Xishanyao Formation in Ili No. 4 Mine in Ili, Xinjiang Province, China. The downhole sampling adopted pump-suction reverse circulation drilling technology to maintain sample intactness to the maximum extent. The sampling position was 138.55 to 147.34 m below the topography, as shown in Figure 1. 

### 2.2. Rock Physics

As indicated by some studies, rock mass permeability is closely correlated to the porosity, cementing form, and sediment percentage [26,27,28,29]. Before laboratorial permeability tests, the paper conducted mercury intrusion tests, scanning electron microscope (SEM, Tescan, Brno, Southern Moravia, Czech Republic) imaging, and X-ray diffraction (XRD, Bruker, Billerica, MA, USA) measurements to obtain WCS porosity, cementation structure, and mineral composition data.

**Figure 2:**
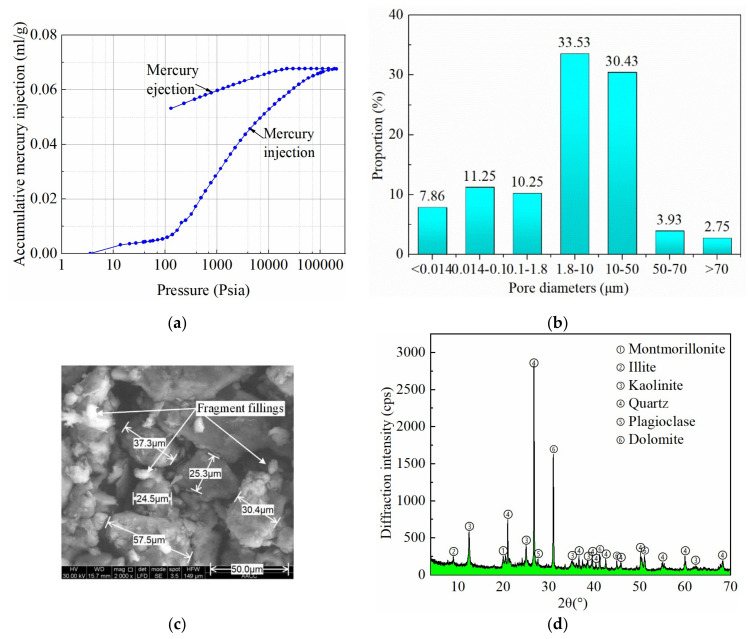
Physical properties of weakly cemented rocks: (**a**) mercury injection–ejection curves; (**b**) pore diameter distribution; (**c**) microscopic structures of pores and minerals via SEM imaging; (**d**) mineral composition via XRD measurement.

Mercury intrusion tests measured WCR porosity of 14.78%. The mercury injection rate of WCR was fast, indicating that the proportion of large-diameter pores in rocks was high. At the same time, the mercury ejection rate of WCR was slow, indicating that the pore connectivity of WCR was poor and the pores were mainly closed or partially closed. Rock pores characterize medium diameters: 1.8–10 μm diameter dominated, taking 33.53% of the total amount, followed by 10–50 μm diameter by 30.43%. WCR was mainly composed of intergranular middle pores [30].The mercury injection–ejection curves and porosity distribution are shown in Figure 2a,b, respectively. Figure 2c shows SEM imaging results, indicating that the pores were extensive and partially filled with fragments, compared to which the mineral particles had greater diameters about 25–60 μm and loosely distributed with explicit boundaries. The XRD measurement obtained WCS mineral compositions, as shown in Figure 2d, revealing that quartz, kaolinite, and montmorillonite took 90% of the total mineral content, among which the quartz and kaolinite minerals were about 34.7% and 33.2%, almost equivalent. As indicated by the XRD results, WCS samples were rich in clay minerals including kaolinite, montmorillonite, and illite, with a percentage of about 55.9%. 

## 3. Complete Stress–Strain Permeability Tests

### 3.1. Methods

**Figure 3:**
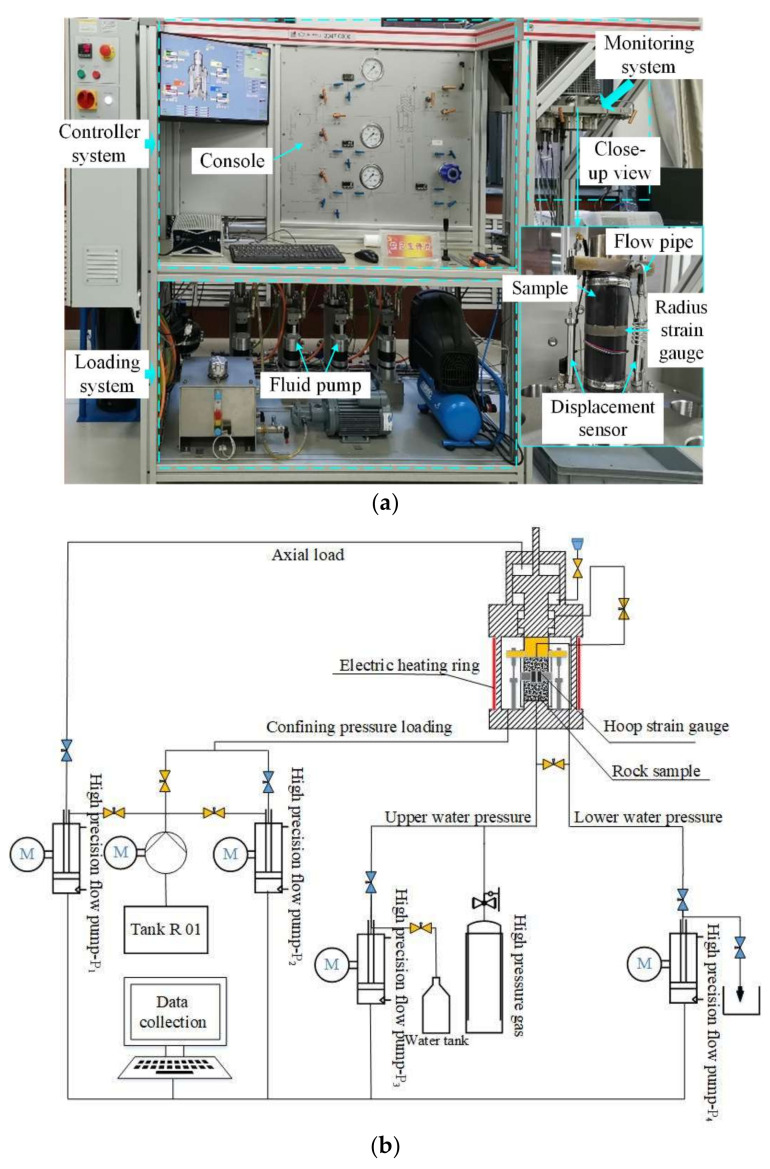
Rock Top multi-physics platform: (**a**) physical picture; (**b**) schematic diagram.

Permeability tests were conducted using the Rock Top multi-physics platform. The platform included a controller system for parameter and servo-control configuration, a hydraulic loading system for executing loading and unloading operations along axial and radius directions, and a monitoring system for sample encapsulation and data (displacement, pressure, and flux) monitoring. The maximum axial stress, maximum confining pressure, and maximum water pressure of the loading system were 500 MPa, 60 MPa, and 50 MPa, respectively. In order to accurately measure the axial deformation and radial strain, two Linear Variable Displacement Transducer (LVDT) displacement sensors were placed in parallel on both sides of the sample with a range of 12 mm and an accuracy of 0.001 mm [31]. Figure 3 exhibits the platform and its three components. The virgin cores were standardized into cylinders with a 100 mm height and 50 mm diameter; the parallelism tolerance of both ends was controlled within 0.1 mm and the diameter difference of both ends were within 0.2 mm. The tests adopted the displacement control method. The samples were saturated using pure water and then tested by keeping the hydraulic pressure difference and loading rate constant. Parameters of the permeability tests as shown in Table 1.

### 3.2. Results

**Figure 4:**
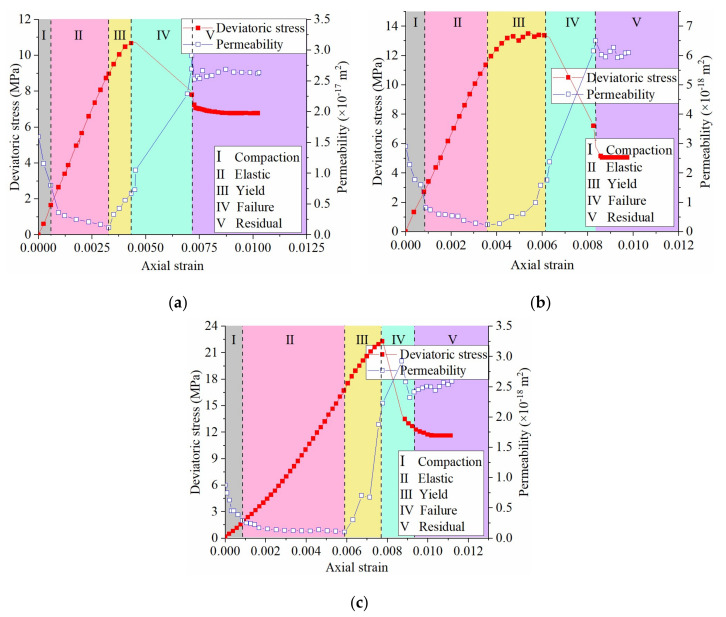
WCS stress–strain–permeability curves under different confining pressures: (**a**) confining pressure 2.0 MPa; (**b**) confining pressure 3.5 MPa; (**c**) confining pressure 5.0 MPa.

**Figure 5:**
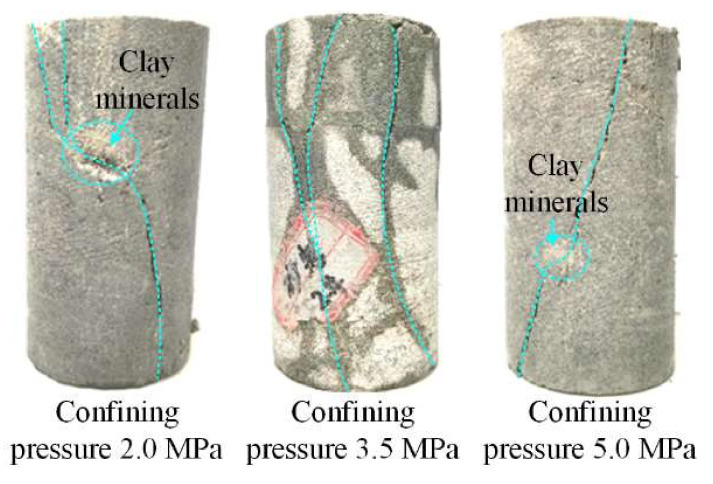
WCS sample failure.

Figure 4 and Figure 5 show WCS stress–strain–permeability curves and sample failure scenarios, respectively.

Figure 4 shows that WCS permeability fluctuated throughout the complete stress–strain stages. The permeability first declined due to rock matrix compression, followed by a further but slight decline to the minimum in the elastic stage. Since rock yielding, the permeability increased and reached the maximum in the middle failure stage. There was again a permeability decline in the later failure stage, after which the permeability was almost unchanged in the residual stage. Of note is that the peak permeability was behind the peak deviatoric stress. An explicit feature of weakly cemented rocks was that the initial permeability (*k*_0_) was relatively high and had a slight difference against the maximum (*k*_max_) and residual permeability (*k*_res_); for example, under the 5 MPa confining pressure condition, the ratio *k*_max_/*k*_0_ and *k*_res_/*k*_0_ was only 3.34 and 2.85, respectively. Table 2 lists the three permeability thresholds.

From Figure 5, it can be seen that the WCS sample failure was mainly characterized by shear fracturing; along fractures there was explicit disintegration of clay minerals.

### 3.3. Relationship between WCR Permeability and Physical Properties

The permeability variation indicated that WCR permeability was closely correlated to the porosity, cementation structure, and mineral composition. The high initial permeability was directly dependent on the high virgin porosity, large pore diameter, and loose connection of mineral particles. The permeability significantly decreased in the compaction stage, practically resulting from the loading-induced compression on extensive pores and thus flow pathways. In the failure stage, the maximum permeability was slightly different than the initial permeability since the abundant clay minerals such as kaolinite and montmorillonite expanded due to encountering water and partially narrowed the fluid flow pathways. As a result, the maximum permeability kept a low level. The residual stage experienced fracture propagation and closure and clay mineral disintegration, thus favoring an almost constant permeability due to a dynamic balance between fresh and existing fractures. In addition, clay minerals took up to 50%, being a major part of the WCR matrix. The clay mineral disintegration suggested that the WCR matrix fractured under the triaxial compression condition.

## 4. Stress–Damage–Permeability Relationship Model

The above analysis indicated that the high porosity, weak cementation structure, and abundant clay minerals caused WCRs to characterize distinctive permeability regimes. Considering that permeability variation is a reflection of rock material damage induced by pressurization, the paper introduced a damage variable *D* to help quantify WCS permeability variation. On this basis, a stress–damage–permeability relationship model was then developed. 

### 4.1. Model Construction

Based on WCS physics and permeability features identified above and existing studies [32], the paper placed the following hypotheses [33,34,35,36].

(1) Weakly cemented rocks were composed of matrix and fractures; the matrix contained natural pores that can be squeezed and closed in the compaction stage and allow matrix materials to be jointed.
(2) The matrix can fracture in response to triaxial compression, further affecting the effective aperture of fractures and thus permeabilities.

**Figure 6:**
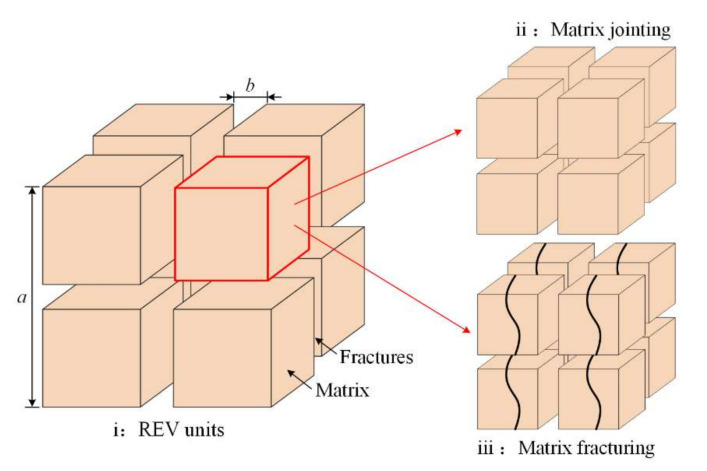
REV description of weakly cemented rocks.

The weakly cemented rocks were described using representative element volume (REV) units as shown in Figure 6.

The REV was the smallest micro element structure that represents the deformation of loaded coal rock mass. Accordingly, the porosity of REV units can be expressed via fracture aperture and matrix geometry [37]:

(1) $$\frac{\phi_{f}}{\phi_{f0}}=\frac{a_{0}}{b_{0}}•\frac{b}{a}=(1+\frac{\Delta b}{b_{0}})\frac{a_{0}}{a}$$

where $\phi_{f}$ and $\phi_{f_{0}}$ are respectively the current and initial porosity of fractures, $a_{0}$ and $b_{0}$ are the initial geometry of matrix and pores, a and b are the current geometry of matrix and pores, and Δb represents the variation of pore geometry.

Existing studies on WCR porosity variation generally placed less focus on the impact of matrix deformation on permeability. In this research, the laboratorial tests revealed that weakly cemented rocks were of high porosity and large pore diameter; the large pores can be geometrically compressed in the compaction stage, and WCR samples well degraded in water. By implication, the paper assumed that the fractured matrix can joint in compressive and fluid flow conditions.

According to this hypothesis, the WCR pore geometry variation Δb was considered composed of matrix geometry change $b_{M}$ and fracture aperture change $b_{F}$, with proportion of $\gamma_{M}$ and $\gamma_{F}$ respectively. Therefore,

(2) $$\left\{ \begin{array}{l} \Delta b=b_{M}+b_{F}\\ \gamma_{M}=b_{M}/\Delta b\\ \gamma_{F}=b_{F}/\Delta b \end{array}\right.$$

where $b_{M}$ and $b_{F}$ are respectively the contribution of matrix and fracture to pore geometry variation, and $\gamma_{M}$ and $\gamma_{F}$ are ratios of the respective contribution.

The volumes of fracture and matrix are:

(3) $$\left\{ \begin{array}{l} V_{F}=a^{2}•b_{F};\;V_{F0}=a_{0}{}^{2}•b_{F0}\\ V_{M}=a^{2}•b_{M};\;V_{F0}=a_{0}{}^{2}•b_{M0} \end{array}\right.$$

where $V_{F}$ and $V_{M}$ are volumes of fracture and matrix, with initial values expressed as $V_{F0}$ and $V_{M0}$, respectively.

The fracture volume and matrix volume have another expression in natural strain and engineering strain [38]:

(4) $$\left\{ \begin{array}{l} V_{F}=V_{F0}\exp(-\frac{\Delta \sigma_{e}}{K_{F}})\\ V_{M}=V_{M0}(1-\frac{\Delta \sigma_{e}}{K_{M}}) \end{array}\right.$$

where $\Delta \sigma_{e}$ is effective stress increment, $K_{F}$ is fracture bulk modulus, and $K_{M}$ is matrix bulk modulus. Considering that the matrix was difficult to compress, this work focused on the influence of $K_{F}$ on the WCR permeability. Meanwhile, the determination of $K_{F}$ was mainly obtained by fitting the stress–strain and permeability curves of WCR.

Combining Equations (2)–(4) obtained the pore geometry variation Δb:

(5) $$\Delta b=(\frac{a_{0}}{a}{)}^{2}\left[b_{F0}\exp(-\frac{\Delta \sigma_{e}}{K_{F}})+b_{M0}(1-\frac{\Delta \sigma_{e}}{K_{M}})\right]$$

The matrix itself was rather rigid but can deform with the contribution of matrix jointing. Consider that the matrix geometry variation obeyed a function of strain $\varepsilon_{e}$, and

(6) $$b_{M}=b_{M0}\frac{a_{0}}{a}(\frac{1}{f(\varepsilon_{e})}{)}^{2}$$

where $\varepsilon_{e}$ is matrix strain and $f(\varepsilon_{e})$ the matrix geometry change, following $f(\varepsilon_{e})$ = $\exp(-\varepsilon_{e})$.

Substituting Equation (6) into Equation (5) obtained:

(7) $$\Delta b=(\frac{a_{0}}{a}{)}^{2}\left[b_{F0}\exp(-\frac{\Delta \sigma_{e}}{K_{F}})+b_{M0}{\left(\frac{1}{f(\varepsilon_{e})}\right)}^{2}\right]$$

Incorporating Equations (1), (2), and (7), with cubic low, obtained WCR permeability expression:

(8) $$k=k_{0}(\frac{a_{0}}{a}{)}^{3}{\left\{1+(\frac{a_{0}}{a}{)}^{2}\left[\gamma_{F}\exp(-\frac{\Delta \sigma_{e}}{K_{F}})+(1-\gamma_{F})(\frac{1}{f(\varepsilon_{e})}{)}^{2}\right]\right\}}^{3}$$

where $k_{0}$ and k are respectively the initial and current permeability.

With continuous pressurization, WCR units damaged and then fractured as the damage gradually accumulated to a certain extent. The fracture initiation and development were accompanied by the matrix geometry variation. By assuming the number of natural fractures and fractures corresponding to the WCR unit damage was respectively $N_{0}$ and N, the matrix geometry variation can be written as:

(9) $$\frac{a_{0}}{a}=\frac{N}{N_{0}}=\frac{N_{0}+\Delta N}{N_{0}}=1+\frac{\Delta N}{N_{0}}=1+D\varsigma$$

where ΔN refers to the fracture number increment, D is damage variable, and ς is the coefficient of matrix geometry variation.

Substituting Equation (9) into Equation (8) obtained a stress–damage–permeability relationship:

(10) $$\begin{array}{l} k=k_{0}\underline{(1+D\varsigma {)}^{3}}{\left\{1+\underline{(1+D\varsigma {)}^{2}}\left[\underline{\gamma_{F}\exp(-\frac{\Delta \sigma_{e}}{K_{F}})}+\underline{(1-\gamma_{F})(\frac{1}{f(\varepsilon_{e})}{)}^{2}}\right]\right\}}^{3}\\ \;\;\;\;\;①\;\;\;\;\;①\;\;\;\;\;②\;\;\;\;\;\;③ \end{array}$$

where items ①, ②, and ③ represent the impact of fracture number, aperture convergence, and matrix joint on permeability, respectively.

### 4.2. Damage Variable Determination

Assume the damage variable *D* obeying Weibull distribution [39]:

(11) $$P\left(F^{*}\right)=\frac{m_{0}}{F_{0}}{\left(\frac{F^{*}}{F_{0}}\right)}^{m_{0}-1}\exp\left[-{\left(\frac{F^{*}}{F_{0}}\right)}^{m_{0}}\right]$$

where $F^{*}$ represents microstructure strength, such as deformation modulus, tensile strength, and cohesion, *m*_0_ is coefficient of average degree, and *F*_0_ is mean value of the strength parameter $F^{*}$.

By Drucker–Prager failure criterion, the microstructure strength $F^{*}$ can be expressed as:

(12) $$\left\{ \begin{array}{l} F^{*}=f\left(\sigma^{*}\right)=\alpha_{0}I_{1}+J_{2}^{1/2}\\ \alpha_{0}=\frac{\sin\varphi }{\sqrt{9+3\sin^{2}\varphi }}\\ I_{1}=\sigma_{1}^{}+\sigma_{2}^{}+\sigma_{3}^{}\\ J_{2}=\frac{1}{6}\left[{\left(\sigma_{1}^{}-\sigma_{2}^{}\right)}^{2}+{\left(\sigma_{2}^{}-\sigma_{3}^{}\right)}^{2}+{\left(\sigma_{1}^{}-\sigma_{3}^{}\right)}^{2}\right] \end{array}\right.$$

where φ is angle of friction, and $\sigma_{1}^{}$, $\sigma_{2}^{}$, and $\sigma_{3}^{}$ refer to three effective principal stress. In this test, the second and third effective principal stresses ($\sigma_{2}^{}$ and $\sigma_{3}^{}$) were consisted equivalent. $I_{1}$ and $J_{2}$ are respectively the first and second invariants of effective stress tensor.

Considering that the damage degree varied with positions, it was obtained via elastic damage theory [33,40]:

(13) $$\left\{ \begin{array}{l} \sigma =E\varepsilon \left(1-C_{n}D\right)\\ D=1-\exp\left[-{\left(\frac{F^{*}}{F_{0}}\right)}^{m_{0}}\right] \end{array}\right.$$

where *E* is deformation modulus, and $C_{n}$ is proportionality coefficient of the damage, reflecting the residual strength of WCR material.

To better reflect WCR mechanics and hydraulics regarding high initial permeability, rapid decline in the compaction stage, and slight change in the residual stage, the overall permeability variation was segmented into compaction elasticity, yielding damage, failure, and residual stages, corresponded to the complete stress–strain profile. Combining Equations (11)–(13) obtained a WCR statistical damage model:

(14) $$\sigma_{1}=\left\{ \begin{array}{l} E\varepsilon_{1}+2\mu \sigma_{3}\;\;\;\;\;\;\;\;\;\varepsilon_{1}\leq \varepsilon_{\mathrm{ela}}\\ E(\varepsilon_{1}-\varepsilon_{\mathrm{ela}})\left(1-C_{n}+C_{n}\exp{\left(-\frac{\frac{E(\varepsilon_{1}-\varepsilon_{\mathrm{ela}})}{\sqrt{3}\left(\sigma_{1}-2\mu \sigma_{3}\right)}\left(\frac{\sin\varphi \left(\sigma_{1}-\sigma_{\mathrm{ela}}+2\sigma_{3}\right)}{\sqrt{3+\sin^{2}\varphi }+\sigma_{1}-\sigma_{3}}\right)+\sigma_{1}-\sigma_{\mathrm{ela}}-\sigma_{3}}{F_{0}}\right)}^{m_{0}}\right)\\ +\sigma_{\mathrm{ela}}+2\mu \sigma_{3}\;\;\;\;\;\;\;\;\varepsilon_{\mathrm{ela}}\leq \varepsilon_{1}\leq \varepsilon_{\max}\\ E{\left(\frac{\varepsilon_{\max}}{\varepsilon_{1}}\right)}^{2}+2\mu \sigma_{3}\;\;\;\;\;\;\varepsilon_{\max}<\varepsilon_{1}\leq \varepsilon_{\mathrm{res}}\;\\ \sigma_{\mathrm{res}}\;\;\;\;\;\;\;\;\;\;\;\;\;\varepsilon_{1}>\varepsilon_{\mathrm{res}} \end{array}\right.$$

where $\varepsilon_{\mathrm{ela}}$ is the strain by the end of the elastic stage, $\varepsilon_{\max}$ is the peak strain, $\varepsilon_{\mathrm{res}}$ is the strain at the start of the residual stage, and $\sigma_{\mathrm{ela}}^{}$ measures the axial stress when axial strain is $\varepsilon_{\mathrm{ela}}^{}$.

Fitting the stress–strain data of each damage stage and residual coefficient determined from residual strength obtained *m*_0_, *F*_0_, *ζ*, and further the damage variable *D* of the WCR stress–strain curves under different confining pressures. Table 3 lists the curve fitting results.

### 4.3. Model Verification

**Figure 7:**
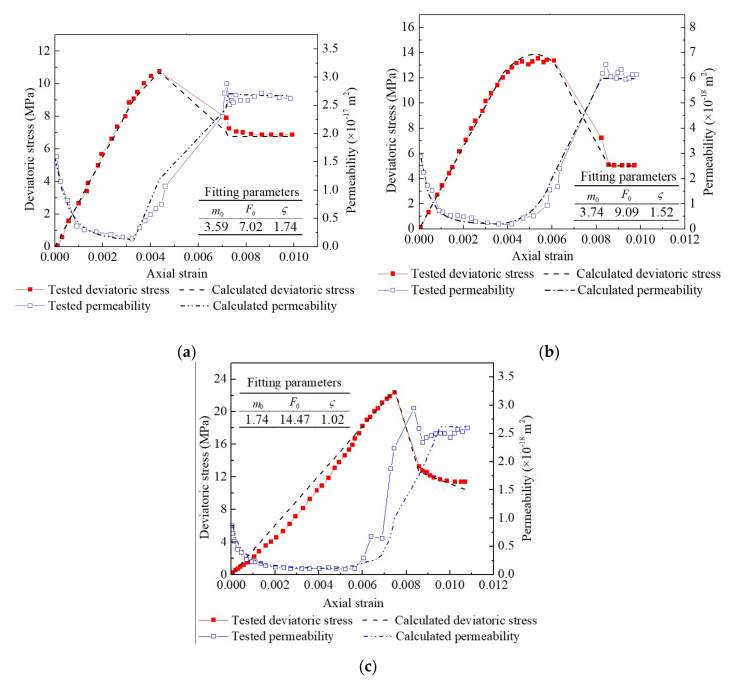
WCR permeability variation curves under different confinement conditions: (**a**) confining pressure 2.0 MPa; (**b**) confining pressure 3.5 MPa; (**c**) confining pressure 5.0 MPa.

To verify the WCR stress–damage–permeability relationship model, the statistical damage parameters summarized in Table 3 were further used to determine the damage variable *D*. Then, the WCR permeability variation under different confinement conditions can be calculated by substituting *D* into Equation (10). The comparison between calculation results and measurement is shown in Figure 7.

Figure 7 suggests a good agreement between theoretical and laboratorial test results, with an error of 12% in deviatoric stress and 15% in permeability, Table 4. This result indicates that the stress–damage–permeability relationship model (Equation (10)) can well describe WCR permeability regime in complete stress–strain stages. 

## 5. Discussion

The WCRs in Jurassic and Cretaceous generally characterize young sedimentation, low strength, and loose cementation. In this work, the Jurassic weakly cemented sandstone is selected from Ili No. 4 Coal Mine in Xinjiang Province, China. The sample selection is very typical and representative. Therefore, the authors believe that the results of permeability test and the established stress–damage–seepage relationship model of WCR based on the typical samples can express its general permeability characteristics. Of course, in the future, the WCRs in more different areas should be tested and fitted to further verify the validity and universal applicability of the established stress–damage–seepage relationship model.

It can be seen from Figure 7c that when the confining pressure is 5.0 MPa, the fitting error of the deviatoric stress and permeability of weakly cemented sandstone is more than 10%. This may be due to the fact that when the confining pressure is 5.0 MPa, the weakly cemented sandstone has obvious elastic brittle failure characteristics (as shown in Figure 7c), and its stress–strain and permeability curves have mutation points (or distortion points). The existence of mutation points destroys the continuity of the curves, which makes its fitting error larger. In future studies, more weakly cemented samples under high confining pressure should be tested and fitted to further explain the above phenomenon.

## 6. Conclusions

Weakly cemented rocks showed permeability fluctuation in the complete stress–strain stages, including a decrease in compaction and elastic segments, an increase in yield and failure segments, and an again decrease to a leveling-off value in the ultimate residual segment. Such a permeability regime was physically correlated to WCR porosity, cementation structure, and mineral composition properties. WCR material had a high initial permeability due to the naturally high porosity, large pore diameter, and loose cementation structure, thus favoring a rapid permeability decline due to pore compression. Since the yield stage, WCR clay minerals such as kaolinite and montmorillonite decomposed into water and narrowed the fractures, causing that the maximum and residual permeabilities were slightly greater than the initial. This minor difference indicated that WCR materials had a limited permeability responses in triaxial confinement conditions, which helps WCR strata maintain a good water-resisting functionality.

XRD measurements revealed that WCR materials were rich in clay minerals. Kaolinite, montmorillonite, and illite minerals took more than 50%, constituting a major part of the WCR matrix. Evident clay mineral disintegration was observed with sample fracturing, implying matrix fracturing in response to triaxial compression.

By assuming that WCR matrix can be compressed, jointed, and fractured, the paper defined a damage variable *D* and further developed a stress–damage–permeability relationship model. Then, a *D* determination method was established according to Weibull distribution and the Drucker–Prager principle. The relationship model was verified with errors in deviatoric stress and permeability within 15% with respect to laboratorial measurements, suggesting that the model can represent the permeability regime of weakly cemented rocks in the complete stress–strain stages.

## Figures and Tables

**Table 1:** Parameters of the permeability tests.

Projects	Confining Pressure (MPa)	Osmotic Pressure by Both Ends (MPa)	Hydraulic Difference (MPa)	Loading Controlled by	Loading Rate (mm/min)
1	2.0	Top: 0.5 Bottom: 1.5	1.0	Displacement	0.03
2	3.5				
3	5.0				

**Table 2:** WCS permeability thresholds (initial, maximum, and residual) in the complete stress–strain stages.

Lithology	Confining Pressure (MPa)	*k*_0_ (m^2^)	*k*_max_ (m^2^)	*k*_res_ (m^2^)	Ratio		
					*k*_max_/*k*_0_	*k*_res_/*k*_0_	*k*_max_/*k*_res_
**Sandstone**	2.0	1.59 × 10^−17^	2.91 × 10^−17^	2.61 × 10^−17^	1.83	1.64	1.11
	3.5	2.91 × 10^−18^	6.50 × 10^−18^	6.06 × 10^−18^	2.23	2.08	1.07
	5.0	8.75 × 10^−19^	2.92 × 10^−18^	2.49 × 10^−18^	3.34	2.85	1.17

**Table 3:** WCR damage parameters.

Lithology	Confining Pressure (MPa)	Statistical Parameters		
		*m*_0_	*F*_0_	*ζ*
**Sandstone**	2.0	3.59	7.02	1.74
	3.5	3.74	9.09	1.52
	5.0	1.74	14.47	1.02

**Table 4:** Error of theoretical WCR deviatoric stress and permeability versus laboratorial measurements.

**Lithology**	**Confining Pressure (MPa)**	**Mean Relative Error (%)**	
		**Deviatoric Stress**	**Permeability**
**Sandstone**	2.0	7.24	7.13
	3.5	5.59	6.32
	5.0	11.39	14.79

## Data Availability

Not applicable.
